# Gene dosage reductions of *Trf1* and/or *Tin2* induce telomere DNA damage and lymphoma formation in aging mice

**DOI:** 10.1038/leu.2015.173

**Published:** 2015-07-31

**Authors:** K Hartmann, A Illing, F Leithäuser, A Baisantry, L Quintanilla-Martinez, K L Rudolph

**Affiliations:** 1 Cooperation Group of the Leibniz Institute for Age Research – Fritz Lipmann Institute (FLI) Jena and Ulm University (UULM), Ulm, Germany; 2 Department of Pathology, University Hospital of Ulm, Ulm, Germany; 3 Institute of Pathology, University Hospital Tübingen, Tübingen, Germany; 4 Faculty of Medicine, Research Group on Molecular Aging, University Hospital Jena (UKJ), Friedrich-Schiller-University (FSU), Jena, Germany

Telomeres are essential structures that cap the end of chromosomes, which is required for maintenance of chromosomal stability, cell viability and the capacity of cells to proliferate. A complex of specific telomere-binding proteins (TRF1, TRF2, POT1, TIN2, TPP1 and RAP1), also known as the Shelterin complex, is essential for telomere capping by assisting the formation of tertiary telomeric structures.^1^ Gene mutations in components of the Shelterin complex (*hTIN2*, *hPOT1* and *hTPP1*) lead to bone marrow failure and cancer formation in human genetic diseases including dyskeratosis congenita (DC), which is caused by Tin2 mutation in 20% of the cases.^2, 3^ All known *TIN2* mutations are heterozygous, autosomal-dominant and patients normally show extremely short telomeres. In addition, mutations in the telomere binding protein *POT1* were shown to lead to lymphocytic leukaemia formation.^4^ Aside from genetic diseases, a variety of studies reported reduced expression of telomere-binding proteins in human cancers compared with non-cancerous tissue suggesting that downregulation of the expression of telomere-binding proteins may also contribute to carcinogenesis in somatic cells and tissues.^5, 6^ It was shown that Epstein–Barr virus-encoded LMP1 and Epstein–Barr virus-infection itself induce the downregulation of TRF1, TRF2 and POT1 at the transcriptional and translational level resulting in complex chromosomal aberrations, alternative lengthening of telomeres and the induction of Hodgkin's lymphoma.^7, 8^

The causal relation between gene dosage reductions of telomere binding protein and the development of cancer and tissue aging remains elusive. Mouse knockout studies revealed that homozygous deletions of *Tin2* or *Trf1* lead to early embryonic lethality.^9, 10^ The conditional homozygous deletion of *Trf1* was shown to provoke severe defects in tissue maintenance^11^ and in combination with homozygous *p53* deletion led to cancer formation in skin.^12^ However, these models did not address the question of whether moderate reductions in the gene dose of telomere-binding proteins contribute to tissue aging and/or carcinogenesis. To address this question we followed aging cohorts of mice carrying heterozygous deletion of *Trf1* and/or *Tin2* in comparison with wild-type mice.^9, 10^

Heterozygous *Trf1*^*+/−*^*Tin2*^*+/−*^ knockout mice showed a 40–50% reduction in the mRNA expression level of Trf1 and Tin2, but had no effect on the mRNA expression profile of other telomere-binding proteins (Figures 1a and b, Supplementary Figures 1a and d). Protein analysis of whole-spleen extracts revealed an ~50% reduced Tin2 expression in *Tin2*^*+/−*^ mice compared with *Tin2*^*+/+*^ mice (Figure 1c, Supplementary Figure 1e). Trusty antibodies for detection of endogenous Trf1 protein in tissues are still lacking. To monitor the Trf1 protein amounts in *Trf1* heterozygous mice, a *Trf1* hemagglutinin (HA)-tag knockin mouse line was generated carrying the HA-tag at the N-terminus of the endogenous *Trf1* gene locus. Opposed to the homozygous *Trf1* knockout mouse, homozygous HA-*Trf1* knockin mice (*Trf1*^ki/ki^) are viable, do not exhibit an overt organismal phenotype and show normal telomere structure indicating that the HA-tag did not interfere with Trf1 function. Trf1 protein from the knockin mice was quantitatively immunoprecipitated with an anti-HA antibody using equally concentrated lysates. Heterozygous *Trf1* deletion led to a reduction in Trf1 protein amounts, whereas the heterozygous deletion of *Tin2* had no significant impact on Trf1 protein levels (Figure 1d; Supplementary Figure 1f). As Trf1 binds directly to Tin2, the heterozygous *Trf1* knockout mice (*Trf1*^*ki/−*^ *Tin2*^*+/+*^) also exhibited a reduction in the amount of Trf1-bound Tin2 compared with *Trf1*^*ki/ki*^ *Tin2*^*+/+*^ mice (Figure 1d; Supplementary Figure 1f).

Immuno-fluorescence *in situ* hybridization staining of telomeres and telomere-binding proteins revealed that heterozygous deletion of *Trf1* reduces the co-localization of Trf1 with telomeric DNA in *Trf1*^+/−^ mice and in *Trf1*^+/−^*Tin2*^+/−^ mice compared with *Trf1*^+/+^ control and *Tin2*^+/−^ mice (Figures 1e and f). Since Trf1 mediates binding of Tin2 at telomeres, heterozygous deletion of *Trf1* also led to a reduced binding of Tin2 protein at telomeres compared to wild-type controls (Figures 1e and g). Heterozygous deletion of *Tin2* reduced Tin2 localization at telomeres compared to wild-type mice but in agreement with the fact that Trf1 binds directly to telomeres *Tin2* deletion did not affect Trf1 expression at telomeres (Figures 1e and f). The heterozygous gene deletions of *Tin2* and/or *Trf1* did not affect the Rap1 localization at telomeres, which is known to bind directly to telomeres in mammalian cells or through its interaction with Trf2 (Supplementary Figures 1g and h). Together, the data on protein expression showed that the heterozygous deletion of *Trf1* and/or *Tin2* lead to reduction of Trf1 and/or Tin2 expression and to reduced localization of the proteins at telomeres in murine cells and tissues.

Cohorts of single heterozygous knockout mice (*Tin2*^+/−^, *n*=21 and *Trf1*^+/−^, *n*=32), double heterozygous knockout mice *Trf1*^+/−^*Tin2*^+/−^ (*n*=33) and wild-type mice (*Tin2*^+/+^, *Trf1*^+/+^, *n*=26) were weekly monitored during aging and exhibited no overt premature aging phenotype (Figure 1h). Analysis of the bone marrow of a cohort of mice at the age of 14–16 months did not reveal evidence for bone marrow failure. Specifically, the *Tin2* and/or *Trf1* gene status did not affect the number of hematopoietic stem and progenitor cells in bone marrow (Figure 1i; Supplementary Figure 1i). Mice were humanly killed when showing >15% of weight loss during aging or other apparent abnormalities (wounds and tumors). Knockout animals exhibited increased incidence of tumors infiltration of spleen, liver and lymph nodes. Kaplan–Meier analysis of tumor-free survival revealed a significant increase in tumor formation in the three cohorts of heterozygous and double-heterozygous knockout mice compared with the wild-type cohort (Figure 1j; Supplementary Figure 1j). Specifically, the analysis indicated that tumor formation in the heterozygous knockout cohorts was accelerated and occurred significantly earlier in life compared with the wild-type cohort.

Overall, 83.3% of the aged wild-type mice were free of tumors when humanly sacrificed due to aging characteristics. Histological analysis of macroscopic tumors revealed that in the wild-type cohort 13.3% of the mice had malignant tumors and 3% carried hyperplastic tumor nodules (Figure 1k). In contrast, 68% (60–76%) of the mice from single or double heterozygous knockout cohorts developed macroscopic tumors including malignant tumors in 54% of the mice (52–56%) and the percentage of hyperplasia was increased to an average of 14% (6.7–20%, Figure 1k). Histological analysis of five representative malignant tumors of *Trf1*^+/−^*Tin2*^+/−^ mice indicated that the majority of the malignant tumors were T- or B-cell lymphoma (Supplementary Figure 1k). Aside from the increased overall frequency, there was no significant shift in the spectrum of tumors in heterozygous or double-heterozygous knockout mice compared with the wild-type cohort.

Studies on telomerase deficient mice indicated that telomere shortening leads to an increase in chromosomal instability and tumor initiation.^13^ To analyze whether such mechanisms were involved in accelerating tumor formation in response to heterozygous deletion of telomere-binding proteins, we studied telomere stability and DNA damage at telomeres in bone marrow derived metaphase spreads of 14–16-month-old mice. Telomere length analysis showed shortening of telomeres from double heterozygous *Trf1*^+/−^*Tin2*^+/−^ mice compared with wild-type mice (Figure 2a). In addition, telomeric fluorescence *in situ* hybridization analysis revealed increases in (i) broken telomeres (Figures 2b and c) and (ii) multi-telomeric signals (Figures 2b and d), both are markers of fragile telomeres and replication stress. Moreover, sister chromatid exchange rates at telomeres (another feature of fragile telomeres) were increased in *Trf1*^+/−^ and *Tin2*^+/−^ as well as in double heterozygous mouse embryonic fibroblasts compared with wild-type mouse embryonic fibroblasts (Figures 2e and f). To analyze whether telomere fragility would also lead to an accumulation of DNA damage, the number of 53BP1 DNA damage foci was analyzed in small intestine of 14–16-month-old mice. There was a significant increase in the number of intestinal epithelial cells in basal crypts harboring 53BP1^+^ DNA damage foci in the heterozygous mutant cohorts compared with control mice (Figures 2g and h). Immunohistochemical staining against phosphorylated H2AX (γH2AX—another marker for DNA breaks) confirmed these results (Figures 2i and j). Co-labeling of telomeres and γH2AX foci revealed a significant increase in telomere-induced DNA damage foci (TIFs) in the heterozygous knockout cohorts compared with wild-type controls (Figures 2k and l).

Together, the current study provides the first experimental evidence that heterozygous gene deletion reduces the binding of Trf1 and Tin2 proteins at telomeres resulting in telomere fragility, DNA damage accumulation and enhanced lymphoma formation in aging mice, but not in premature aging *per se*. These results indicate that a tight control of the expression level of telomere-binding proteins is important to avoid tumor formation, but premature failure in organ homeostasis of patients carrying heterozygous *TIN2* mutations likely involves gain of function or dominant negative effects of the mutant alleles as drivers of organ failure. Recent findings on reduced expression of telomere-binding proteins, telomere uncapping and increases in sister chromatid exchange rates in human lymphoma and in response to Epstein–Barr virus infection of human B-lymphocytes suggest that the results from this study are relevant for the development of human hematopoietic malignancies.^7, 14, 15^ Alterations in the expression level of telomere-binding proteins could represent an alternative, telomere length independent route to telomere dysfunction, which in turn induces hematopoietic malignancies.

## Figures and Tables

**Figure 1:**
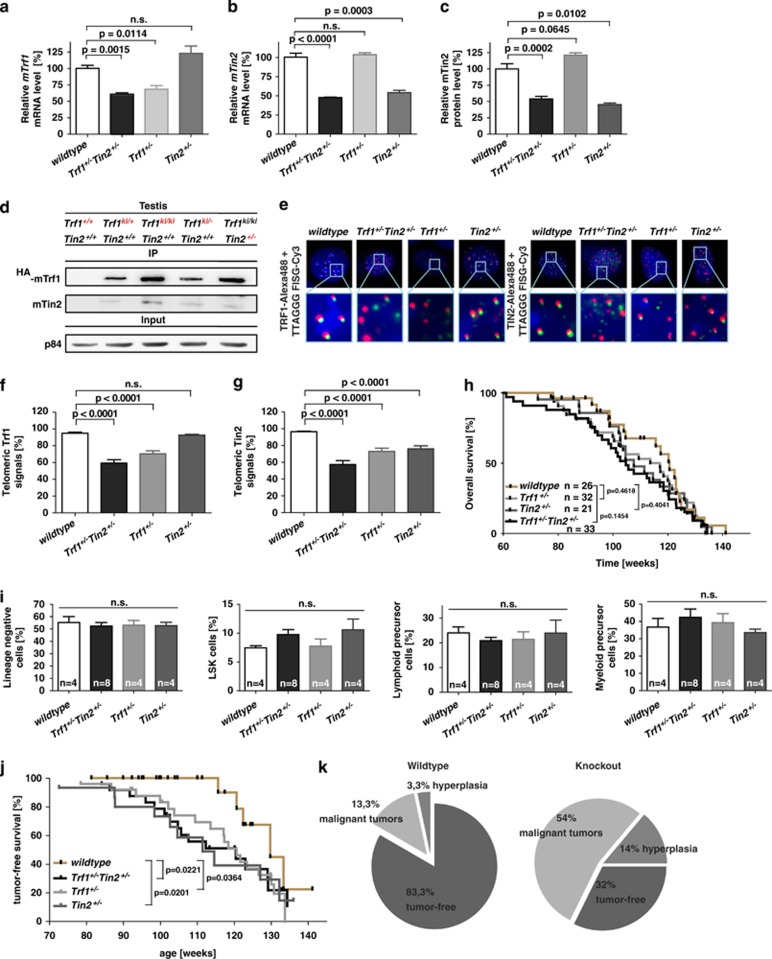
Heterozygous loss of *Trf1* and *Tin2* leads to a dose-dependent reduction in expression, affects their binding to telomeres and leads to the enhanced formation of tumors during aging. (**a** and **b**) Quantitative analysis of *Trf1* (**a**) and *Tin2* (**b**) mRNA levels normalized to hydroxymethylbilane synthase in total spleen extracts of 14–16-month-old mice of the indicated genotypes. (**c**) Quantification of Tin2 protein expression levels relative to GAPDH by western blot analysis in total spleen protein extracts of 14–16-month-old mice of the indicated genotypes. (**d**) *Trf1*^+/−^ and *Tin2*^+/−^ mice were crossed with HA-tagged *Trf1* knockin mice (*Trf1*^*ki/ki*^). Western blot detection of a N-terminal HA-tagged version of Trf1 (^HA^mTrf1) and mTin2 after immunoprecipitation of ^HA^Trf1 with an HA-specific antibody from protein extracts of testis of mice of the indicated genotypes. Western blot analysis of p84 was used as loading control. (**e**) Immuno-fluorescence *in situ* hybridization (FISH) staining of telomere-binding proteins (Trf1 and Tin2) and telomeric ends in mouse embryonic fibroblasts (MEFs) of the indicated genotypes. FISH of telomeric repeats was conducted using a [TTAGGG]_3_-Cy3 peptide nucleic acid (PNA) probe (red). Specific antibodies against the Shelterin proteins were used in combination with a secondary Alexa-488 labeled antibody (green). (**f** and **g**) Percentage of co-localization of Trf1 (**f**) and Tin2 (**g**) foci at telomeric ends from MEFs of the indicated genotypes. (**h**) Kaplan–Meyer curves showing survival of wild-type mice (*n*=26), *Tin2*^+/−^ mice (*n*=21), *Trf1*^+/−^ mice (*n*=32) and *Trf1*^+/−^*Tin2*^+/−^ mice (*n*=33). The overall survival rate of the indicated genotypes is not significantly affected during the observation period of 140 weeks. (**i**) Quantification of the hematopoietic stem and progenitor cell compartment in 14–16-month-old mice by fluorescence activated cell sorting of the indicated genotypes. The percentages were calculated for viable in total bone marrow (panel I–III) (*n*=4–8 mice per group). (**j**) Kaplan-Meyer curves showing tumor-free survival of the indicated mouse cohorts. Note that the heterozygous deletion of *Trf1* and/or *Tin2* reduced the latency of tumor formation. 18–24-month-old *Trf1*^+/−^ mice, *Tin2*^+/−^ mice and *Trf1*^+/−^*Tin2*^+/−^ mice showed increased rates of tumor formation compared with wild-type mice developing tumors at 24–30 month. (**k**) Spectrum of the histological analyzed tumors. Malignant tumor formation is significantly enhanced in the knockout mice compared with the wild-type mice. Error bars indicate s.d. and the Student *t*-test was used for statistical calculations. (**h** and **j**) the Mantel–Cox test was used for calculations of *P*-values.

**Figure 2:**
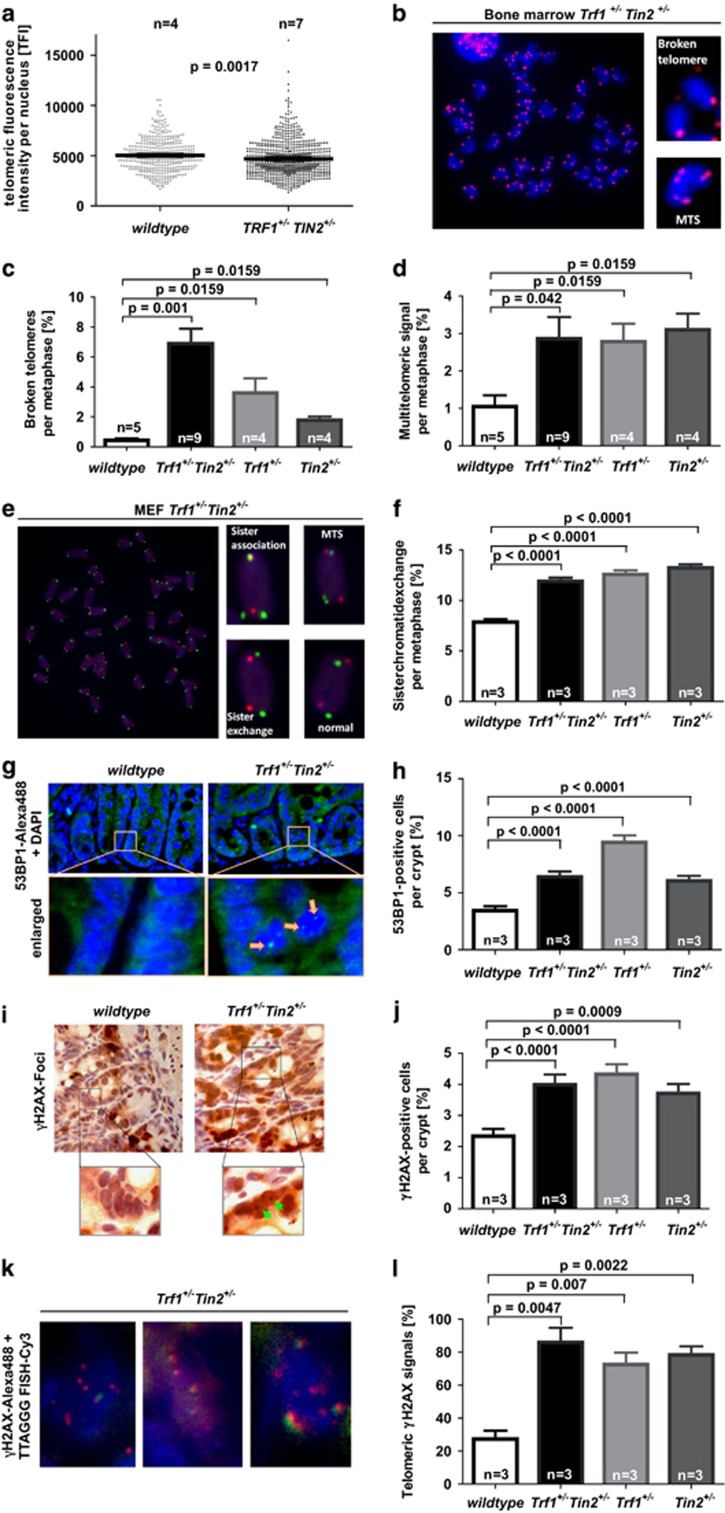
*Trf1* and *Tin2* deficiency leads to increased telomere fragility and sister chromatid exchange accompanied by an accumulation of DNA damage at telomeric ends *in vivo*. Metaphase spreads from freshly isolated bone marrow cells were analyzed by fluorescence *in situ* hybridization (FISH) using telomere specific probes: (**a**) the telomere fluorescence intensity (TFI) was quantified from 410 cells of wild-type mice and 728 cells of *Trf1*^+/−^*Tin2*^+/−^ mice. The dot plot shows the mean TFI of individual nuclei. (**b**) Representative images of telomere fluorescence (Q-FISH) distribution in metaphase spreads from *Trf1*^+/−^ *Tin2*^+/−^ mice using a [TTAGGG]_3_-Cy3 peptide nucleic acid (PNA) probe. High magnifications on the right show telomere fragility characterized by multitelomeric signals (lower picture) or broken telomeres (upper picture). (**c** and **d**) Quantification of the percentage of broken telomeres (**c**) and multitelomeric signals (**d**) per metaphase from bone marrow cells of the indicated genotypes. 144 individual metaphases of wild-type mice, 294 metaphases of *Trf1*^+/−^*Tin2*^+/−^ mice, 204 metaphases of *Trf1*^+/−^ mice and 236 metaphases of *Tin2*^+/−^ mice have been analyzed. (**e**) Metaphase spreads from *Trf1*^+/−^ *Tin2*^+/−^ mouse embryonic fibroblasts (MEFs) were analyzed by telomeric CO-FISH using a [TTAGGG]_3_-Alexa488 PNA probe (green) and a [CCCTAA]_3_-Cy3 PNA probe (red). High magnifications on the right show telomere fragility characterized by chromosomes with sister chromatid fusions (upper left), multitelomeric signals (upper right) or sister chromatid exchange (lower left). (**f**) Frequency of sister chromatid exchanges in metaphase spreads of MEFs from the indicated genotypes. 297 individual metaphases of wild-type mice, 316 metaphases of *Trf1*^+/−^*Tin2*^+/−^ mice, 301 metaphases of *Trf1*^+/−^ mice and 307 metaphases of *Tin2*^+/−^ mice have been analyzed. (**g** and **h**) Analysis of 53BP1 foci in the basal crypts of the small intestine from 14–16-month-old mice of the indicated genotypes: (**g**) representative images of 53BP1 foci in the basal crypts, (**h**) percentage of cells showing 53BP1 signals in the nuclei of crypt cells. In total, cells from 185 crypts of wild-type mice, 229 crypts of *Trf1*^+/−^*Tin2*^+/−^ mice, 149 crypts of *Trf1*^+/−^ mice and 146 crypts of *Tin2*^+/−^ mice have been analyzed. (**i** and **j**) Analysis of γ-H2AX foci in the basal crypts of the small intestine from 14–16-month-old mice of the indicated genotypes: (**i**) representative images of γ-H2AX foci in the basal crypts, (**j**) percentage of cells showing γ-H2AX signals in the nuclei of crypt cells. In total, cells from 244 crypts of wild-type mice, 230 crypts of *Trf1*^+/−^*Tin2*^+/−^ mice, 231 crypts of *Trf1*^+/−^ mice and 245 crypts of *Tin2*^+/−^ mice have been analyzed. (**k** and **l**) Immuno-FISH analysis of γ-H2AX foci at telomeres using a γH2AX antibody (green) and a [TTAGGG]_3_-Cy3 PNA probe (red): (**k**) representative images of γH2AXat telomeres in crypts of *Trf1*^+/−^ *Tin2*^+/−^ knockout mice, (**l**) percentage of cells with co-localization of γ-H2AX foci at telomeres. *n*=number of independent MEF cultures used per genotype. Error bars indicate s.d. and *P*-values are indicated. (**c**, **d**, **f** and **j**) the Mann–Withney test was used for calculations of *P*-values. (**h** and **l**) The Student *t*-test was used for calculations of *P*-values.
