# Erratum: Gene dosage reductions of *Trf1* and/or *Tin2* induce telomere DNA damage and lymphoma formation in aging mice

**DOI:** 10.1038/leu.2017.224

**Published:** 2017-11-10

**Authors:** K Hartmann, A Illing, F Leithäuser, A Baisantry, L Quintanilla-Martinez, K L Rudolph

**Keywords:** Telomeres, DNA damage response, DNA-binding proteins, Lymphoma

**Correction to:** *Leukemia* (2016) **30,** 749–753; doi:10.1038/leu.2015.173; published online 31 July 2015

Following the publication of this article the authors noted that the article was lacking the information on n-numbers for some of the panels of the figures.

The description of the results section is specified for the following information on *n*-numbers of mice: The western blots in Figure 1D and Supplementary Figure 1F were conducted as biological replicates (testis and thymus) using pools of mice of the indicated genotypes (*n*=6 mice per genotype for thymus, *n*=4 mice per genotype for testis). The quantification of the expression of telomere binding proteins was conducted on RNA level (Figure 1a and b; Supplementary Figure 1A–D, *n*=3 mice per genotype) and on protein level (Figure 1c; Supplementary Figure 1E, wild type: *n*=6 mice, Trf1+/−Tin2+/−: *n*=7 mice, Trf1+/− mice: *n*=4 mice, Tin2+/−: *n*=4 mice). In the latter analysis, one of the total seven wild-type mice was excluded due to an oversaturated GAPDH signal by statistical outlayer analysis (Grubbs’ test). The quantification of the telomeric signals of telomere binding proteins (Figure 1f and g; Supplementary Figure 1H) was carried out on a total of 37–43 nuclei per genotype from three independent cultures of mouse embryonic fibroblasts. The tumor-free survival curves (Figure 1j) and the pie chart on tumor formation (Figure 1k) were calculated from the identical number of mice as shown in Supplementary Figure 1J. For Figure 1k all mice from the three different knockout cohorts were combined.

The authors wish to apologize for any inconvenience caused.

