# Supplementary figures and images for: Gene dosage reductions of Trf1 and/or Tin2 induce telomere DNA damage and lymphoma formation in aging mice
Source: Leukemia. 2015 Jul 31;30(3):749–53. doi: 10.1038/leu.2015.173 (PMC4777776; doi:10.1038/leu.2015.173)

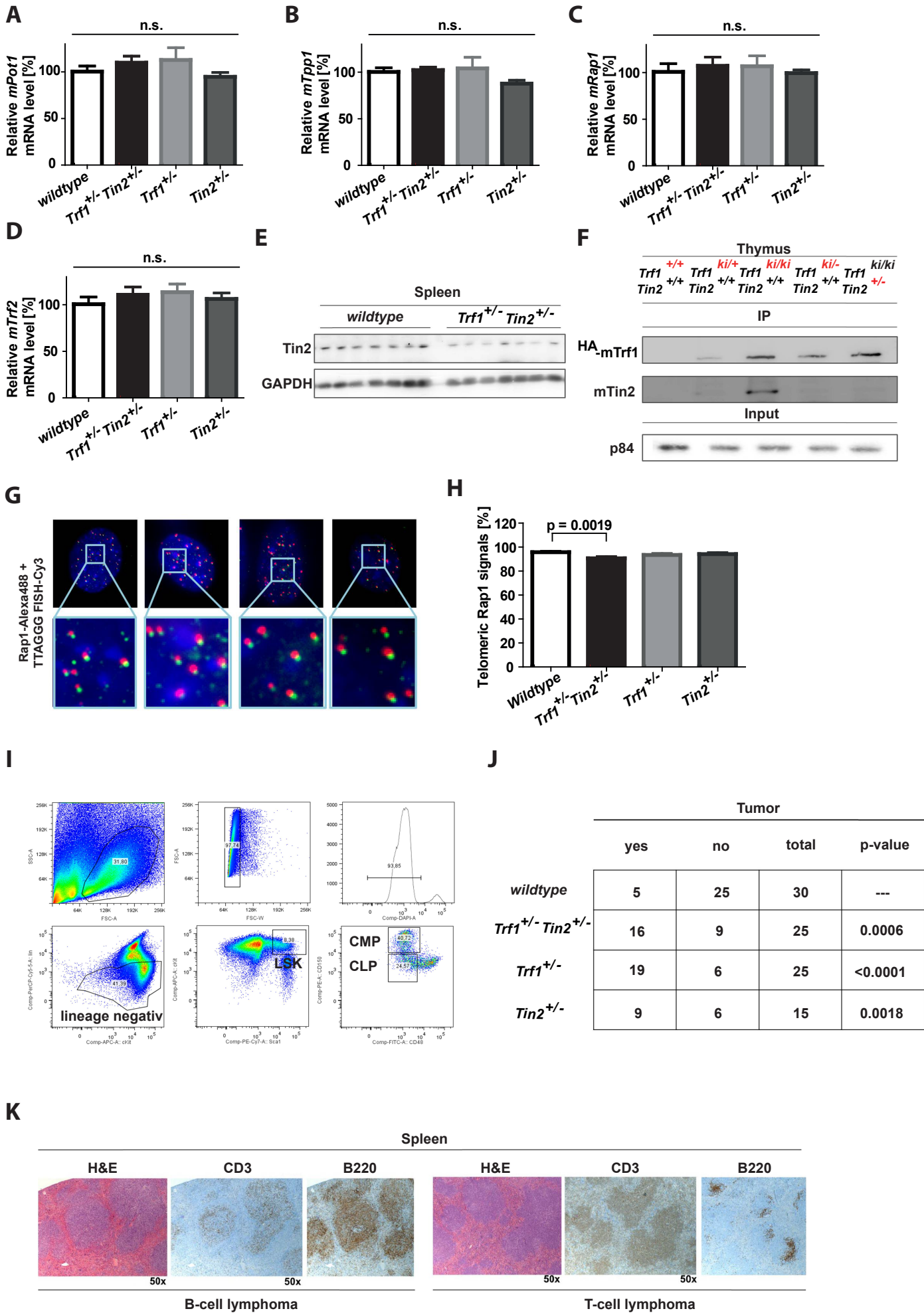

Supplement: Supplementary Figure 1 [file leu2015173x1.pdf]
