# Supplementary material for: Gene dosage reductions of Trf1 and/or Tin2 induce telomere DNA damage and lymphoma formation in aging mice
Source: Leukemia. 2015 Jul 31;30(3):749–53. doi: 10.1038/leu.2015.173 (PMC4777776; doi:10.1038/leu.2015.173)
Supplement: Supplementary Information [file leu2015173x2.doc]

Supplementary Information

**Supplementary Figure 1**: **Analysis of Shelterin components after heterozygous loss of *Trf1* and *Tin2* and histological analysis of the tumor spectrum of single and double heterozygous mice.**

(**A-D**) Quantitative analysis of mRNA expression levels *Pot1* (**A**)*, Tpp1* (**B**)*, Rap1* (**C**) and *Trf2* (**D**) in total spleen extracts of 14-16 month old mice of the indicated genotypes. (**E**) Representative Western Blot for quantitative analysis of TIN2 protein expression levels. (**F**) *Trf1^+/-^* and *Tin2^+/-^* mice were crossed with HA-tagged *Trf1* knockin mice (*Trf1*^ki/ki^). Western Blot detection of a N-terminal HA-tagged version of Trf1 (^HA^mTrf1) and Tin2 after immunoprecipitation of ^HA^Trf1 with an HA-specific antibody from protein extracts of thymus of mice of the indicated genotypes. Western Blot analysis of p84 was used as loading control. (**G, H**) Immuno-FISH staining of Rap1 and telomeric ends in mouse embryonic fibroblasts (MEFs) of the indicated genotypes (**G**). FISH of telomeric repeats was conducted using a [TTAGGG]_3_-Cy3 PNA probe (red). Specific antibody against Rap1 was used in combination with a secondary Alexa-488 labeled antibody (green). (**H**) Percentage of co-localization of Rap1 foci at telomeric ends from mouse embryonic fibroblasts of the indicated genotypes. (**I**) Representative FACS analysis of the hematopoietic stem and progenitor cell compartment in 14-16 month old mice by fluorescence activated cell sorting (FACS). (**J**) The diagram shows the overall tumor-incidence in mice of the indicated genotypes analyzed at an average age of 107.6 weeks (*Tin2^+/-^* mice), 115.4 weeks (*Trf1^+/-^* mice), 104.7 weeks (*Trf1^+/-^Tin2^+/-^* mice) and 120.6 weeks (*wildtype* mice). (**K**) Representative images of haematoxylin & eosin (H&E), B220- and CD3-stainings from spleens of *Trf1^+/-^Tin2^+/-^* mice that either show a B-cell lymphoma or a T-cell lymphoma.

Error bars indicate standard deviation. The Student´s *t*-test was used for statistical calculations and *p*-values are indicated.
